# Editorial: Machine Learning Techniques on Gene Function Prediction Volume II

**DOI:** 10.3389/fgene.2022.949285

**Published:** 2022-06-30

**Authors:** Ren Qi, Arun Kumar Sangaiah, Dariusz Mrozek, Quan Zou

**Affiliations:** ^1^ Yangtze Delta Region Institute (Quzhou), University of Electronic Science and Technology of China, Quzhou, China; ^2^ School of Life Science and Technology, University of Electronic Science and Technology of China, Chengdu, China; ^3^ School of Computing Science and Engineering, VIT University, Vellore, India; ^4^ Institute of Informatics, Silesian University of Technology, Gliwice, Poland; ^5^ Institute of Fundamental and Frontier Sciences, University of Electronic Science and Technology of China, Chengdu, China

**Keywords:** machine learning, genetics, bioinformatics, feature selection, deep learning, single cell sequencing data

Predicting the function of genes is a critical problem in biology. The current generation rate of new gene sequences is too fast to discover and validate them experimentally, emphasizing the importance of machine learning. Machine learning techniques have advanced our understanding of gene function, which have been widely employed to study amongst other things the interaction among genes and proteins, diseases and differentiation. The power of a combination of machine learning and biological analysis can be found in our first installment, Machine Learning Techniques on Gene Function Prediction Volume I, especially in predicting gene and ncRNA function.

We believe it is not the end, so we planned the second special issue on this subject, Machine Learning Techniques on Gene Function Prediction Volume II. In the first installment, we found most authors paid attention to gene and ncRNA function prediction. This Research topic will further explore the potential for machine learning applied to gene function prediction. Moreover, we would also like to share some works on single-cell sequencing data analysis and related machine learning methods. We are pleased to receive many submissions with the new sight of machine learning techniques combined with gene function prediction. All of these papers were accepted for publication with the assistance of professional referees. Twenty-four papers are finally selected from all submissions after rigorous reviews.

There are seven papers describing protein function prediction or protein identification. Ma et al. identified Soluble N-ethylmaleimide sensitive factor activating protein receptor (SNARE) proteins based on iLearnPlus and solved the problem of data imbalance. Coincidentally, Zhang et al. proposed a machine learning method to recognize SNARE proteins based on SVM and improved the identification accuracy compared with existing methods. In addition, Wan et al. distinguished immunoglobulins and non-immunoglobulins by FC* and GC* features, where immunoglobulins are critical in disease regulation. To identify hormone-binding proteins (HBPs), which are important to organisms’ growth, Guo et al. present a prediction model HBP_NB, combining with k-mer feature representation, feature selection, and Naive Bayes. Furthermore, Gong et al. developed a machine learning method to identify vesicular transport proteins. Besides protein recognition, there are still two papers on protein function prediction. Li et al. presented a new multi-label classifier to explore protein function by embedding multi-type features. Meanwhile, Chen et al. provided a computing method that combined knowledge of the protein-protein interaction network and functional characteristics to help predict human protein subcellular localization patterns and their potential biological importance. Chien et al. paid attention to gene expression prediction in T- DNA mutants through machine learning methods on rice functional gene research.

With a wide range of mathematical statistics capabilities, composable machine learning methods can help automate and analyze complex relationships between genes and disease. Three papers focused on this issue in general diseases, which contain heart disease, Alzheimer’s disease, and androgenic alopecia. Wang et al. predicted the occurrence of heart failure (HF) events in hemodialysis (HD) patients by the extreme gradient boosting method. To overcome the curse of dimensionality on Alzheimer’s disease gene expression datasets, Wan et al. designed a hybrid gene selection pipeline combined with deep learning methods to improve the classification of this disease. Li et al. focused on mental stress recognition of depression disorders in patients with androgenic alopecia (AGA), and they analyzed the effect of psychological interventions in the rehabilitation of AGA patients by machine learning and fuzzy K-means clustering method FAW-FS, which combined with metaheuristic, the Filter and Wrapper algorithms. Li et al. created MIMRDA to classify top-ranked miRNAs. The method incorporated miRNA and mRNA expression profiles to predict associations between miRNA and disease and identify key miRNAs and recommended potential biomarkers as well. Two papers reviewed machine learning and deep learning methods applied in disease-gene related. Gong et al. discussed the research progress of lncRNAs and reviewed disease-related lncRNA methods and the relationships between lncRNAs and diseases. Fang et al. reviewed machine learning and deep learning methods for Ischemic Stroke disease.

Five papers focus on discussing the gene and cancer relationship. Chen et al. provided a computing method based on machine learning to predict anticancer peptides (ACPs), which is very important for the discovery of new cancer treatment drugs. Huo et al. employed dysregulated networks (DNs) to analyze subtypes of breast cancer, they measured the regulation strength between genes based on gene expression values. Huang et al. explored the function of DEG between lung cancer tissues and revealed the molecular driving mechanism of lung cancer. Chen et al. concentrated on Lung squamous cell carcinoma (LUSC) study, they screened key factors that regulate the initiation and progression of this disease by a metric learning analysis method. In addition, Han et al. reviewed sparse representation methods’ applications in bioinformatics, such as cancer molecules and gene expression profiles fields.

Another aspect is studying the interactions between gene and protein, gene and gene, protein and protein. DNA-protein interactions, such as gene expression and transcriptional regulation, are tightly linked to DNA-binding proteins (DBP), Jia et al. proposed a feature extraction method that fused multiple PSSM features to predict DBP. To deepen the understanding of the principle of protein interactions, Tang et al. proposed a new hierarchical attention network structure HANPPIS predicting protein-protein interaction sites. For the study of gene-gene interactions (GGIs), most methods are based on assumptions about GGIs forms. To improve statistical performance, Guo et al. tested GGIs based on a maximal neighborhood coefficient perspective in Genome-wide association studies, which outperformed other baseline methods. Besides, Xu et al. reviewed drug-target interaction and specific applications of machine learning technique prediction methods. Notably, our special issue showed a new sight on multi-omics data storage and parallel processing. Mrozek et al. gave a large-scale and serverless computational approach for improving the quality of NGS data supporting big Multi- Omics data analyses.

To conclude, papers in this special issue have demonstrated the power of machine learning techniques in a broad range of gene function studies, especially in inferring the relationships between genes and diseases. We highly expect such studies will get great attention. Especially, more insightful results are desirable for promoting the development and progress of biology. Finally, we thank all efforts of the authors, reviewers, and staff at the Frontiers in Genetics editorial office.

